# Messaging preferences among Florida caregivers participating in focus groups who had not yet accepted the HPV vaccine for their 11- to 12-year-old child

**DOI:** 10.1186/s12889-022-14852-9

**Published:** 2022-12-22

**Authors:** Stephanie A. S. Staras, Carma L. Bylund, Michaela D. Mullis, Lindsay A. Thompson, Jaclyn M. Hall, Marta D. Hansen, Carla L. Fisher

**Affiliations:** 1 Department of Health Outcomes and Biomedical Informatics, College of Medicine, University of Florida, 2004 Mowry Road, Gainesville, FL 32610 USA; 2 Institute for Child Health Policy, University of Florida, 2004 Mowry Road, Gainesville, FL 32610 USA; 3 Department of Advertising, College of Journalism and Communications, University of Florida, 2096 Weimer Hall 1885 Stadium Rd, PO BOX 118400, Gainesville, FL 32611 USA; 4 Department of Pediatrics, College of Medicine, University of Florida, 1600 SW Archer Rd, Gainesville, FL 32610 USA

**Keywords:** Vaccine hesitancy, HPV vaccination, adolescents, clinician recommendations, reminders

## Abstract

**Background:**

In the United States, human papillomavirus **(**HPV) vaccination rates remain low. The President’s Cancer Panel suggests that effective messaging about the HPV vaccination focus on the vaccine’s safety, efficacy, ability to prevent cancer, and recommendation at ages 11- to 12-years. We aimed to develop messages about HPV vaccine that include the President Cancer Panel’s suggestions and were acceptable to caregivers of adolescents.

**Methods:**

From August to October 2020, we conducted one-hour, Zoom videoconference focus groups with caregivers who lived in Florida, had an 11- to 12-year-old child, and had not had any of their children receive the HPV vaccine. Focus group moderators asked caregivers to react to three videos of clinician (i.e., MD, DO, APRN, PA) recommendations and three text message reminders. Thematic analysis was conducted using the constant comparative method and led by one author with qualitative analysis expertise. Two additional authors validated findings.

**Results:**

Caregivers (*n* = 25 in six groups) were primarily non-Hispanic white (84%) and educated (64% had at least an Associate’s degree). Approximately a third of caregivers had delayed (44%) or decided against a vaccine for their child (36%). Caregivers described six preferred message approaches: recognize caregivers’ autonomy, balanced benefits and risks, trustworthy sources, increased feasibility of appointment scheduling, information prior to decision point, and preferred personalized information. Caregivers expressed a desire to have the follow-up doses mentioned in the introduction.

**Conclusions:**

HPV vaccine messages, whether delivered by a clinician or via text message, will be more acceptable to caregivers if they approach HPV vaccination as the caregivers’ decision, and include information from trusted sources to help caregivers make an informed choice.

## Background

Human papillomavirus (HPV) vaccination has the potential to prevent 73% of HPV-related cancers including cervical, oropharyngeal, and vaginal cancers [1]. In the United States (US), completion of all doses of HPV vaccination recommended by the US Centers for Disease Control and Prevention’s Advisory Committee on Immunization Practices for all 11- to 12-year-olds remains low (59%) compared to other high-income counties (e.g., 80% girls and 74% boys in Australia) [2–6]. Unlike at least 95 countries worldwide, including Australia, Sweden, and Great Britain, the US does not have a national school immunization program [7, 8]. While specific schools have implemented vaccination programs, adolescent vaccines have not been routinely offered in schools and the primary location of vaccine distribution in US is at private or public clinics [7, 9]. Enhancing clinician (i.e., MD, DO, APRN, PA) recommendations and sending reminders to caregivers are established effective approaches to increase HPV vaccination [10–14]. The most salient content for these messages to maximize caregivers’ acceptance and completion of the HPV vaccine series remains uncertain.

Clinician recommendations and discussion of the HPV vaccine with caregivers are associated with 10- to 11-fold increases in vaccine initiation among their children [15]. Studies observing clinicians in real-world settings suggest that presumptive approaches to discussing the vaccines, such as mentioning the vaccines are due or will be given, are more effective than starting open-ended conversations [16–20]. One study trained clinicians to use a presumptive approach for recommending the HPV vaccine to parents of adolescents, called the Announcement Approach, announcing that three vaccines are due with the HPV vaccine in the middle of the list; the training was tested in a general pediatric population and increased HPV initiation but not up to date rates [16].

To increase up to date rates, it is likely necessary to move HPV vaccine acceptance in response to the Announcement Approach from passive acceptance to a decision. Studies consistently show that vaccine accepting and hesitant parents have safety concerns about vaccines including the fear of long-lasting side-effects and limited risk of infection [21]. Vaccine hesitant parents report appreciating their child’s clinician explaining the risks and benefits of vaccines and demonstrating vaccine-related expertise [22]. Thus, especially for vaccine hesitant parents, it may be important for clinicians to balance a strong recommendation with parents’ desire to receive balanced information [21]. A presumptive approach that includes additional information on the importance and safety of receiving the HPV vaccine may be more effective for increasing up to date rates while maintaining the strategy’s speed and effectiveness.

In addition to clinician recommendations, there is overwhelming evidence supporting reminder/recall as an evidence-based strategy [10, 11, 14, 23]. Reminders for the HPV vaccine typically state that the child is due or overdue for the HPV vaccine and sometimes includes other due vaccines, an option to schedule an appointment, or education content [10, 14, 24]. We previously tested HPV vaccine reminders that included the President’s Cancer Panel recommended components and found they were acceptable to caregivers and increased HPV vaccine initiation [24, 25]. Prior evidence suggests that creators of health messages should consider adding emojis or weblinks to additional information [25, 26].

As part of a larger intervention to evaluate the possible synergistic effect of text messages and clinician recommendations, we conducted focus groups with caregivers of 11- to 12-year-olds living in Florida. Our main aim was to identify message content that caregivers prefer when receiving HPV vaccination information from clinicians or via text messages. Our secondary aim was to assess the acceptability of various aspects of text messages (i.e., inclusion of emojis and weblinks to additional information) [25–27]. We structured recommendation and text message content around the President’s Cancer Panel recommended topics (importance of receiving the vaccine at age 11- to 12-years, vaccine safety, and the ability of the vaccine to prevent cancer) [28, 29].

## Methods

### Design and Procedures

From August 5 to October 20, 2020, we conducted one-hour focus groups with the primary caregivers of 11- to 12-year-olds residing in Florida who had not accepted the HPV vaccine for any child. These restrictions reflected our aim to reach caregivers of adolescents within the Centers for Disease Control and Preventions’ (CDC) recommended age for universal coverage (11–12 years) who were considering the vaccine [2]. To participate, we required that caregivers’ express willingness to receive text messages and photos from the study team on their cell phone during the group discussion. We also restricted participants by geography: initially restricting participation to caregivers reporting residing in Florida counties within the bottom third of HPV vaccine initiation rates statewide [30]. To speed recruitment, we expanded eligibility to counties below the state median (August 24) and to all Florida counties (September 29), and increased participant incentives from $30 to $60 (August 24). The study was approved by the University of Florida Institutional Review Board.

We aimed to conduct 5 focus groups of 4–10 caregivers each because evidence suggests that main themes will be present with as few as 12 participants and 2–3 groups [31, 32]. Similarly, groups of as few as 3 participants may exhibit active discussion and produce rich data [33]. Caregivers were recruited via Facebook with paid advertisements and posts on the University of Florida’s Research Studies webpage. In response to study staff requests, several moderators of Florida-based, parent special interest Facebook groups reposted the study. The advertisements included stock photos of adolescents (boys and girls from various racial/ethnic groups) alone or with a mother or father figure and a link to a University of Florida’s Research Studies webpage that contained a description of the study’s key components with a link to a survey in REDCap, an online, secure survey tool, that included the above screening criteria (age of child, children who had received the HPV vaccine, county of residence, interest in online group discussion, and willingness to receive text messages during the focus group).

Caregivers who met the screening criteria were invited to consent and complete a background survey in REDCap that included questions related to vaccination adapted or adopted from previous surveys [30, 34–42]. Focus groups were conducted by Zoom videoconference because the study occurred during the COVID-19 pandemic prior to vaccine availability. Focus group audio recordings were professionally transcribed.

Two trained focus group moderators used a semi-structured interview script to solicit caregivers’ perspectives. The interview script was developed following best practices to create a comfortable environment that would allow parents to express their opinions on key features of the messages [43]. The interview script was pilot tested with health-related students and research staff to improve conversational flow and participant comprehension. In all groups, text messages were discussed before clinician recommendations because focus group best practices include starting questioning with easier to reflect upon content and this sequence is consistent with how parents will encounter the information in real-life. Within the category of text message or clinician recommendation, messages were presented in a random order for each group to reduce priming effects.

#### Text messages

Each text message included the three recommended components by the President’s Cancer Panel and personalization to the child’s first name (Table 1) [28, 29]. Based on caregivers’ opinions that educational postcards had insufficient information [25], two messages included web links to additional vaccine information; however, we varied the source of the links to assess preferences [27]. Based on the importance of emojis in text messaging [26], two messages included emojis. All messages prompted caregivers to respond affirmatively that they would like to have the child’s doctor’s office call them [10, 44].

**Table 1:** Clinician recommendations presented on video during focus groups

Today, your child will receive a vaccine that protects against cancer for both boys and girls. It is important that kids get this vaccine at ages 11 or 12 years.	
We have Gardasil for Nico. This vaccine prevents cancer, is safe, and it is important that kids get it at ages 11–12 years, so he’ll get the first one today and you’ll come back to get the second dose in 6 to 12 months.	
We have a vaccine that prevents against six types of cancer. Now that Sara is 11 years old, I recommend that she get this safe vaccine today.	

Moderators instructed caregivers to imagine that the text message was received two weeks prior to their child’s 11th birthday. Once caregivers received text messages, they were asked to rate how likely (five-point scale) they would be to respond Yes. The moderator summarized the ratings and led a discussion focusing on the following: message layout, anything not making sense, and suggested changes. When relevant, caregivers were asked about their reaction to Gardasil, Happy Birthday, emojis, and weblinks.

#### Clinician recommendations

Three clinician recommendations were presented by video of a white, female practicing pediatrician (name, degree, and title displayed) speaking to the camera in a clinical setting. We chose this individual because she represents the most prevalence demographic of pediatricians (66% white and 71% female) and she has a compassionate demeanor [45, 46]. Clinician introductory recommendations included a presumptive approach and 2–3 of the President’s Cancer Panel’s recommended components (Table 2) [28, 29]. After each video, we assessed caregivers’ likelihood of agreeing (five-category scale) to have their child receive the HPV vaccine that day. The moderator summarized the group’s responses and asked questions about: likes and dislikes, factors the stood out, anything not making sense, suggested changes, and response to presumptive statement. Once all three clinician videos were discussed, caregivers were asked about their overall reactions.

**Table 2:** Text messages sent to caregivers during focus groups

Did you know you can protect <child’s_first_name> from certain cancers? The HPV vaccine is safe, free and most useful if received at ages 11 or 12 years. To learn more, go to www.cdc.gov/hpv/parents. Reply YES to have <child’s_first_name>‘s doctor’s office call you.	
Have you heard about the Gardasil vaccine? It is safe, free, and a vaccine that can protect against certain types of cancer. Now that <child’s_first_name> is 11, you can help protect <him/her>! Reply YES to have <child’s_first_name>‘s doctor’s office call you to schedule an appointment.	
Happy 11th birthday to <child’s_first_name>! Now that <he/she> is 11, <he/she> can receive a safe and effective vaccine that can protect <him/her> from HPV cancers. To learn more, go to https://ufhealth.org/hpv-vaccine. Reply YES to have <child’s_first_name>‘s doctor’s office call you to schedule an appointment.	
Notes: (1) All messages included: “Reply STOP to opt-out. Msg&data rates may apply” (2) Each message was personalized with the 11- to 12-year-old child’s first name	

### Data Analysis

Using a constant comparative approach and data management software (ATLAS.ti), a thematic analysis was led by a qualitative expert (CF) and validated by two coders (CB, MM) trained in thematic analysis (and not involved in data collection) to increase rigor [47, 48]. All authors reviewed the finalized codebook adding expertise in HPV vaccination implementation, adolescent primary care, and the interpretations of focus group moderators. The analytical approach is informed by an interpretivist paradigm and involves systematic steps to approach the data inductively using open and axial coding [27]. Analysis involved 3 steps: 1) assigning codes (i.e., labels) to identify concepts in the text, 2) collapsing codes into categories to identify themes (which were message approaches), and 3) conducting axial coding (i.e., finding patterns identified within the data specific to each theme) to isolate properties of themes that characterize each [49]. The same themes were often identified in both contexts (text versus clinician communication). Repetition (repeated similar words to describe the same phenomenon), recurrence (using different terms to describe the same phenomenon), and forcefulness (emphasis) were the standard and inter-related criteria used to ensure thematic saturation [50]. Best practices in focus group methodology were also used by ensuring saturation was obtained across groups and participants [33]. All focus groups were included in the analysis because they exhibited active discussion and had rich data [33]. Both themes and properties of each theme emerged in at least 2 focus groups, with most found in 3–5 focus groups. Themes were reported by at least 43% of participants, with most reported by between 63 and 95% of participants. Excerpts are labeled by focus group and participant (e.g., FG1, P1).

## Results

Twenty-five caregivers of 11- to 12-year-olds participated in 6 focus groups with 3–7 participants each (demographics in Table 3). Caregivers described six preferred message approaches (i.e., themes) to clinician communication and text messages. By conducting an additional level of analysis (axial coding) we also captured properties of themes (noted below in italics), the emergent patterns that define each theme. These properties illustrate reasons for preferences, how approaches informed decision making, and strategies to enact approaches. Themes are presented as action statements in Table 4 to promote translation to practice [51–53].

**Table 3:** Characteristics of participating caregivers of 11- to 12-year-olds (*n* = 25)

Characteristic	N	%
Relationship to youngest 11-to 12-year-old		
Biologic mother	23	92%
Biologic father	1	4%
Aunt	1	4%
Race/Ethnicity		
Non-Hispanic White	21	88%
Hispanic	1	4%
Non-Hispanic African-American	1	4%
Non-Hispanic African-American and White	1	4%
Highest level of education		
Master’s or Doctorate	7	28%
Bachelor’s	7	28%
Associate Degree	2	8%
Some College	7	28%
Vocational school or High school	2	8%
Marital status		
Married or unmarried and living with partner	20	80%
Divorced or separated	5	20%
Number of 11- to 17-year-old children		
1	14	56%
2	8	32%
3–4	2	8%
RUCC for county of residence		
Metro areas of 1 million population or more	11	44%
Metro areas of 250,000 to 1 million population	13	52%
Nonmetro-Urban population 2500 to 19,999, adjacent to a metro area	1	4%
Region of residence		
Northwest	1	4%
Northeast	14	56%
Central	4	16%
Suncoast	3	12%
Southeast	3	12%
Ever delayed a shot for youngest 11- to 12-year-old child for reason other than illness or allergy		
Yes	11	44%
No	14	56%
Ever decided not to get a vaccine for youngest 11- to 12-year-old child for reason other than illness or allergy		
Yes	9	36%
No	16	64%
Gender of youngest 11- to 12-year-old child		
Female	16	64%
Male	9	36%
Likelihood that your youngest 11- to 12-year-old child will receive the HPV vaccine within the next year		
Very likely	2	8%
Somewhat likely	8	32%
Not too likely	4	16%
Don’t know / Unsure	11	44%

**Table 4:** Preferred message approaches to receiving HPV vaccine information

Caregivers want messages that	From this approach, messages should
Recognize caregivers’ autonomy	Recognize caregivers’ autonomy by inviting questions (e.g., don’t tell them what to do; facilitate shared decision making by asking parent for their questions) Clarify this is the clinician’s recommendation and caregivers’ choice (e.g., respect caregivers’ options and control; add credibility)
Attend to specific questions that inform decision making	Address the pros and cons (e.g., reduces cancer risk; side effects) Explain why it’s important for boys and girls (e.g., tailor to the patient) Identify the timeframe and age span for administration (e.g., help caregivers plan and understand urgency) Include information about the history of the vaccine (e.g., evidence of safety and efficacy) Clarify that it requires two doses (e.g., help caregivers plan and understand immunity)
Utilize trustworthy sources (with text messaging)	Identify it was from their child’s healthcare clinician’s office (e.g., recognized name and number) Eliminate characteristics that convey a “sales pitch” (e.g., some emojis, brand name of vaccine) Incorporate informational links from experts (e.g., CDC or community source)
Address logistical feasibility	Include a reminder of well-visit appointment (e.g., text message clarifying vaccine done at the same time) Include an option to easily schedule (e.g., text message with links to schedule)
Provide information well ahead of time	Give caregivers time to research and prepare (e.g., provide messages with links to sources to research on own and come with questions) Allow caregivers time to address what can be a challenging topic for the first time (e.g., given it’s about sexual health; not feel caught off guard)
Use personalized information	Draw caregivers in, making it more likely they would respond (e.g., grab their attention with a birthday message, child’s name) Foster caregivers’ sense of personal connection (e.g., make them feel remembered and cared for)

### Theme 1: Recognize Caregivers’ Autonomy

Caregivers preferred that clinicians acknowledge their “options” by facilitating dialogue versus strong presumptive clinician recommendations and text messages, which were perceived as “forward” and “aggressive.” Caregivers wanted clinicians to invite questions to open communication*.* Caregivers didn’t want to feel “pressured” or “forced” and favored “a more open conversation”:

I was extremely off put by [doctor saying] “Today your child will be getting this.” This should be a conversation, not telling me we’re getting anything. … Even if I came here wanting a vaccine I would be really put off. … Some people don’t want to feel stupid, like they should already know these things so they’re not going to ask the doctor … They don’t want to feel like they’re, usurping the doctor’s authority in some way or questioning her recommendations. … I would love for her to end with “What questions do you have?” Something that is inviting of a little bit more conversation. (FG1, P2).

Caregivers wanted introductory messaging to explicitly acknowledge their choice and clinician’s recommendation*.* The vaccine should be “presented as an opportunity rather than being told” (FG3, P1). Hearing the clinician “recommended” it added “credibility.” Caregivers also linked “choice” and “recommendation” with facilitating discussion:

[Say] “This is what we recommend, and this is why we recommend it, and when you make a choice, we’re here to support you in your choice.” That seems very much like a conversation I would have at my pediatrician’s office. … It shows parents have a choice. … Once you take that choice away … it feels like “us versus them.” (FG6, P3).

### Theme 2: Attend to Specific Questions That Inform Decision Making

Caregivers had questions they wanted answered, which they linked to promoting vaccine uptake. They want to address the pros (e.g., reducing the risk of specific types of cancer) and cons (e.g., side effects)*:* “I want to know both sides. … I can’t imagine a scenario in which I would agree to a vaccine without having pros and cons “(FG3, P1). Caregivers also wanted tailored messages to explain its importance for boys and girls*,* which could dispel misconceptions: “Have messages for girls and messages for boys, since they affect them differently. I would’ve had no idea that this was for boys” (FG3, P2). Additionally, caregivers wanted messages to identify the timeframe and age span for administration with supporting rationale, which informed their “sense of urgency” and ability to plan: “I really like where it says it’s most useful if received at ages 11 or 12. … It gives you sort of a timeframe … Maybe I should jump on that” (FG1, P2). Messages should also include the history of the vaccine with evidence demonstrating it was “safe” and “effective” (e.g., “How long [its] been around? What follow-up studies have been done?” (FG6, P1)). This was critical as caregivers viewed the vaccine as “new”: “Educate us about what it is that we’re vaccinating against. It’s a new vaccine. It’s not something that when I was a kid we had. … Are these kids being guinea pigs for this?” (FG2, P2). Finally, caregivers wanted messages to clarify vaccination requires two doses to enhance planning. It should “recommend that you come back … to follow up and do the second dose” (FG5, P3). This also promoted caregiver’s understanding of efficacy, that two doses were needed for “building up immunity.”

### Theme 3: Utilize Trustworthy Sources

Especially for text messages, caregivers wanted to know the information was from a trusted source. Caregivers wanted messages to identify the source—the clinician’s office by showing their name and number. It was vital that caregivers had previously received texts from their child’s pediatrician (or an awareness they might receive texts from the clinic):

I’d be alarmed to seeing my son’s name on there … [Add] something that has where it came from specifically … With the way things are right now—with the scams and the kid trafficking and all this stuff. (FG2, P3).

Caregivers suggested messages eliminate characteristics that convey a “sales pitch” (emojis, vaccine’s brand name). Caregivers associated these features with spam or suspicions about pharmaceutical companies: “The brand name of the vaccine—that came up a little bit like a commercial. … It’s like disingenuous. Is the doctor trying to help me? Are they getting a kickback from the vaccine manufacturer?” (FG1, P2). Lastly, the message should incorporate informational links from “nonpartisan” or “balanced” sources: “If it came from the manufacturer of the vaccine, I would find that very biased. … When it came from the CDC, then I take that as a pretty solid source” (FG6, P3). Caregivers in one focus group indicated distrust of the CDC, preferring a local health institution source: “Before COVID, the CDC would be better. But now, CDC can be kind of polarizing to different people” (FG2, P1).

### Theme 4: Address Logistical Feasibility

Caregivers liked messages promoting vaccination feasibility. They wanted messages to include a reminder of an upcoming (or needed) well visit appointment, noting the vaccine could be administered then. Reminders could clarify that “you can do this as part of a regular annual well check instead of making it sound like it’s a stand-alone appointment” (FG1, P2). Caregivers preferred reminders include an option to easily schedule (e.g., the office could call them):

As a busy mom, telling me that I have to remember one more thing, is sort of just adding to my list of things to do. That’s really helpful. … I can just say “yes” and somebody will call me and remind me to make an appointment. (FG6, P3).

### Theme 5: Provide Information Well Ahead of Time

Caregivers wanted information early, even a year prior, suggesting it become “standard practice to mention it at the 10-year-old appointments.” By getting information early caregivers have time to research and prepare*.* Caregivers felt “blindsided” getting information when the vaccination was to be administered. Caregivers suggested information be sent ahead of time to prepare for the appointment: “That’s going to make you feel informed to be able to make that decision right then” (FG5, P2). Having information early helped caregivers have time to address a challenging topic (sexual activity), which was “awkward” for everyone:

I felt very caught off guard when I took my son in and they asked me if I wanted a vaccine to prevent cervical cancer. … My misconception was that it was something for females. … [I said] “He’s a boy. He doesn’t have a cervix. Why would he need that?” [Doctor] whispers, really uncomfortably, “In case he has oral sex.” I said, “Oh.” My son’s sitting [there]. He said, “Mommy, what did she say?” … I tell her “You tell him.” She says, “Well, no.” She was embarrassed. We never went any further with it. (FG3, P2).

### Theme 6: Use Personalized Information

Caregivers liked text messages that were personalized with the child’s name, birthday, or age. Caregivers believed this approach could draw caregivers in, promoting a response*.* Caregivers found messages “wishing my child a happy birthday” appealing and trustworthy. “I would respond to the text. … The fact that my child’s name is spelled correctly … tells me that it’s not spam” (FG5, P4). Personalized information could also foster caregivers’ sense of connection*.* Caregivers felt cared for and “remembered”: “It was personal, more personal. I would feel ... ‘Oh, how nice of her! She was thinking about him!’” (FG3, P2).

## Discussion

Caregivers of 11- to 12-year-olds who had not yet vaccinated any of their children for HPV found their child’s clinic sending of text messages about HPV vaccine acceptable, identified preferred message approaches, and gave suggestions to enhance the messages’ acceptability. Caregivers felt some of the presumptive wording compromised their autonomy and wanted messages to include the need for a second dose. For text messages, caregivers desired to establish that the information was from a trusted source and valued the timing of just prior to the child’s 11th birthday. Incorporating caregivers’ preferences for HPV vaccine messages could enhance caregivers’ satisfaction and ultimately increase vaccination rates.

Florida caregivers participating in our focus groups had strong preferences for recognition of their autonomy in the decision making. Caregivers were supportive of the simple introductory statements, but similar to prior qualitative research with vaccine hesitant parents about vaccines wanted the clinician to acknowledge the parent’s choice [54]. Our finding of caregiver dislike of strong presumptive statements is consistent with prior research showing that caregivers are less confident about their vaccine decisions when clinicians express that the decision to vaccinate is urgent and the prevalent themes in social media that suggest parents “do your own research” [55–57]. It is, however, important to consider this finding from focus groups within the context of recording studies of real-world clinician recommendations that demonstrate caregivers who received presumptive recommendations rather than non-presumptive recommendations perceived the recommendation as very strong, had less concerns about safety, and were less likely to remain hesitant about the HPV vaccine [58]. Additionally, clinician recommendations that emphasize parent choice have been shown as less effective [59]. Therefore, similar to a proposed framework for vaccine recommendations [60], caregivers who express vaccine hesitancy may require more information than provided in the Announcement Approach to address their concerns and future research should consider what message wording will strongly recommend the vaccine while acknowledging caregivers’ autonomy [61]. Furthermore, increased understanding of caregivers’ cognitive processing when making vaccine decision for their child could aid message development. While not tested, this could possibly be achieved by softening the language (e.g., changing “your child will receive a vaccine” to “I recommend your child receive a vaccine”) or adding an open-ended question like “What questions do you have?”. Alternatively, following the presumptive statement with easing caregivers’ concerns may be sufficient [56, 62]. These nuanced modifications may become even more important as clinicians address potentially increased vaccine hesitancy since the beginning of the COVID-19 pandemic [63].

Caregivers’ preferred messages included the need for and timing of the second dose because it allowed them to anticipate and plan for the next steps. Our finding is consistent with increased up to date rates when text messages include the timing of subsequent doses [64]. Moreover, clinicians’ recommendation of the HPV vaccine is associated with increased up to date rates [15], but there is little evidence regarding what provider message content maximizes up to date rates.

Consistent with American society’s growing distrust of the media and caregivers’ concerns with child online safety [65, 66], caregivers wanted to verify the text message was from a trusted source. Trust is a central component of healthcare and effective vaccine communication [67]. Suggested technical solutions to increase trust in the message focused on clarifying that the text message was from their child’s doctor by including the clinic’s name. Some caregivers described having greater trust in the local university’s health center than the CDC, which might reflect this community’s deterioration of trust in this national health agency related to the COVID-19 pandemic and might be contemporary and time-limited [68]. Trust in government health agencies, however, historically varies by demographics and information-seeking behaviors [27]. Taken together with caregivers’ desire for balanced information in the clinician recommendation, our findings on caregivers wanting to confirm information sources suggest that caregivers may enter vaccine conversations with skepticism.

Caregivers’ interpretation of text messages sent near the child’s birthday as advanced notice supports prior findings where reminder messages primed caregivers to accept the HPV vaccine during preventive visits [25]. Caregivers’ desire for more time to consider the vaccine is consistent with Leask’s classification of late or selective vaccinators [60], and may support the practice of clinicians integrating HPV vaccine discussions into 9- or 10-year-old visits as preparatory [69]. Expanding prior findings of boosting caregivers’ confidence about the HPV vaccine when their clinician included the vaccine’s recommended ages [56], caregivers felt messages that included recommended ages enabled them to fit the decision-making process into their schedules.

The study has three notable limitations. First, clinician recommendations were presented via video by a clinician unknown to the caregivers. Thus, the setting, context, and personal trust with their child’s clinician differ from real-world clinical care. While only some families have a trusting relationship with their child’s clinician [70], caregiver responses may not reflect how they would respond to their child’s clinician. Second, the study over-represented non-Hispanic whites compared to Florida’s population (53% in Florida versus 84% in our study) [71]. Third, while our relatively small sample (*n* = 25) produced notably saturated findings consistent with prior studies, results might not reflect caregivers in Florida or other states, especially since compared to the general population, an increased percentage of caregivers in our study had delayed (7% versus 44%) or refused (5% versus 36%) a vaccine [38].

The main strength of this study is that we evaluated caregivers’ reactions to text message and clinician delivered HPV vaccination recommendations following a widely suggested presumptive recommendation approach that included the three key constructs suggested by the President’s Cancer Panel [16–20, 28, 29]. Our study differed from a randomized trial that popularized the presumptive approach for HPV vaccine recommendations among the general population by focusing on caregivers of 11- to 12-year-olds who had not yet accepted the HPV vaccine, whom a large percentage indicated some hesitancy to the vaccine, and who live in a state where HPV vaccination rates are below the national average [16, 40]. While effective in a randomized controlled trial [16], caregivers, especially caregivers who are uncertain about the HPV vaccine, may not appreciate the authoritarian sentiment of the presumptive approach to vaccine recommendations.

## Conclusion

Caregivers had strong preferences regarding clinician recommendations and text messages that if incorporated into communication efforts might help increase HPV vaccination rates. Most importantly, caregivers felt that presumptive recommendations limited their opportunity for questions and threatened their autonomy. Caregivers wanted complete and balanced information from a trusted source and expressed a need for a schedule of future doses presented in the initial recommendation. HPV vaccine messages, whether delivered by a clinician during an office visit or via text message, may be more acceptable to caregivers if they recognize their autonomy, provide a timeline for the decision-making process, and include information from trusted sources.

## Data Availability

The data that support the findings of this study are available on request from the corresponding author [SS] with a signed data use agreement. The data are not publicly available due to the small sample from one clinic potentially compromising the research participant privacy.

## Abbreviation

HPV: Human Papillomavirus
